# Effect of selenium supplementation on lipid profile in hemodialysis patients

**DOI:** 10.15171/jrip.2016.38

**Published:** 2016-05-30

**Authors:** Hamidreza Omrani, Sima Golmohamadi, Yahya Pasdar, Kambiz Jasemi, Afshin Almasi

**Affiliations:** ^1^Nephrology and Urology Research Center, Kermanshah University of Medical Sciences, Kermanshah, Iran; ^2^Department of Nutrition, Kermanshah University of Medical Sciences, Kermanshah, Iran; ^3^Department of Internal Medicine, Kermanshah University of Medical Sciences, Kermanshah, Iran; ^4^Department of Biostatistics and Epidemiology, Kermanshah University of Medical Sciences, Kermanshah, Iran

**Keywords:** Chronic kidney disease, Hemodialysis, Selenium, Lipid, End stage renal disease

## Abstract

**Introduction:** One of the major causes of mortality in chronic kidney disease (CKD) patients on hemodialysis is premature atherosclerosis. Selenium, a trace element involved in important enzymatic activities inside the body, has protective effects against lipid oxidation and inhibits cholesterol accumulation in blood vessels.

**Objectives:** To determine the effect of selenium supplementation on lipid profile in hemodialysis patients.

Patients and Methods: In this double-blinded randomized clinical trial which lasted for 3 months, 84 hemodialysis patients with selenium deficiency were divided into experimental group (received selenium supplementation) or control group (received placebo). Total cholesterol, low-density lipoprotein (LDL-C), high-density lipoprotein (HDL-C), blood urea nitrogen (BUN), creatinine, and selenium level were measured before and after the study.

**Results:** Mean (±SD) serum LDL-C level significantly increased in experimental group from 85.66 (±31.12) to 109.12 (±32.29) mg/dl (*P*<0.001). Likewise, in control group serum LDL-C significantly increased from 80.55 (±21.13) to 97.05 (±28.07) mg/dl (*P*<0.001). However, with control of LDL-C effect before and after the study, it was revealed that LDL-C change was not statistically significant (*P*=0.21). Similarly, total cholesterol and triglyceride levels did not show significant changes before and after the study in any group.

**Conclusion:** Selenium supplementation had no beneficial effect on lipid profile in hemodialysis patients.

**Implication for health policy/practice/research/medical education:**

Since there is controversy about selenium supplementation on lipid profile in patients with hemodialysis patients, we conducted a study to determine the role of omega-3 in such patients. Our results showed that in short-term follow-up selenium supplementation, selenium supplementation had no beneficial effect on lipid profile in hemodialysis patients.

## Introduction

The premature atherosclerosis is one of the main causes of death among hemodialysis patients. Dyslipidemia is the main cause of atherosclerosis development in such patients (1,2). Some studies have suggested that hemodialysis per se causes increased production of free radicals (3-5). It is likely that some small proteins such as immunoglobulin G (IgG) and complement system elements adhere to the membrane of dialysis system and cause granulocyte activation which in turn causes production of free radicals (1,2,6). Hence, increased lipid peroxidation as well as antioxidant depletion could be the contributing factors in atherosclerosis in hemodialysis patients (1,6,7).

Recently, the role of lipid peroxidation products has drowned widespread attention in the pathogenesis of atherosclerosis (7). Oxidative stress, as a result of excessive production of reactive oxygen species (ROS) by activated monocytes and depression of antioxidant system, exists in chronic kidney disease (CKD) patients. This oxidative stress facilitates the process of atherogenesis in CKD patients. A growing body of evidence indicates a decrease in antioxidant function for the elimination of ROS in hemodialysis patients (1,2). Among the most important antioxidant defense systems of body are glutathione peroxide (GSH-PX) and superoxide dismutase (SOD) enzymes which their performances require copper, zinc and selenium.

Selenium is a trace element and is an essential component of GSH-PX (Ec1.11.1.9) (8). Selenium is an important trace mineral in the human body. This nutrient is an essential part of enzymes of the body’s cells against free radicals in which by participating in the structure of GSH-PX will protect them. Free radicals are formed during natural oxygen metabolism process of the body and have widespread destructive effects on the body. Among essential functions of selenium in the body which can also be cited is the role it plays in preventing both heart disease and elevated blood pressure (9,10). Selenium by protecting lipid inhibits its oxidation, in particular LDL, which in turn prevents cholesterol deposition in arterial wall (11).

Chen et al (12) compared 81 hemodialysis patients with 42 healthy individuals and found out that selenium level in patients with chronic renal failure is significantly lower than in the healthy individuals, and consequently, the high prevalence of selenium deficiency and increased levels of cadmium have been observed in patients with renal failure. In another report, it was shown that serum selenium level in hemodialysis patients was significantly lower than in control group, and there was a negative correlation between selenium level and uric acid which may involve the risk of heart disease (13). Fujishima et al in a study conducted on 1041 patients, who were divided according to their levels of serum selenium into four groups, concluded that serum level of selenium has an inverse relationship with the risks of death among patients, particularly risk of death as a result of infectious disease among hemodialysis patients, and any decreased serum level of selenium can be a contributing factor in reducing the levels of safety, and it may increase the risk of death from infectious disease in hemodialysis patients (14).

Since micronutrients are important components in metabolism pathways of proteins and enzymes and have many metabolic functions, any changes in their serum level can have profound impact on other important and critical parts of the body. In view of the fact that antioxidant activity is lowered in patients with CKD, we decided to evaluate the role of selenium as an effective factor in antioxidant activity of these patients. Since such a study has never been conducted in Iran, and no effort has been made in previous studies in attempting to homogenize the two groups and eliminate potential risk factors, this research attempts to study hemodialysis patients and determine the levels of serum of selenium and its impact on patient’s biochemical parameters for the purposes of reducing mortality and morbidity of cardiovascular (given that cardiovascular disease is the most common cause of mortality in these patients as taken a positive step in reducing complications in hemodialysis patients).

## Patients and Methods

In this double-blinded clinical trial, the study population consisted of all patients who presented to out tertiary care center to undergo hemodialysis and has passed 6 months from hemodialysis initiation. Exclusion criteria were viral hepatitis or other infectious diseases, taking steroidal and non-steroidal anti-inflammatory drugs, taking of vitamins E and C during the two preceding months prior to the study, and having normal serum level of selenium.

The sampling method was of convenience method and sample size was calculated as 42 patients in each group using selenium level and data of a previous study (15) considering confidence level of 99% and power of 95%.

Before connection to hemodialysis machine, 3 cc of venous blood was obtained and the following markers were measured: complete blood count (CBC), total cholesterol, low-density lipoprotein (LDL), high-density lipoprotein (HDL), blood urea nitrogen (BUN), creatinine, and selenium level.

The patients were randomly divided (using random number table) into experimental and control groups. Experimental group received selenium capsules for three months, while control group received placebo (starch) capsules. After three months, the above mentioned laboratory markers were measured again. After completion of hemodialysis, weight with light clothing using a weight scale with precision of 1 kg was measured. Height was also measured without shoes by a meter installed on the wall with precision of 1 cm.

In cases where lipid abnormality was severe and the patients required multi-dimensional treatments (diet, supplements, and medication), necessary treatments were delivered after completion of the study under supervision of nephrologist.

### Ethical issues

The research followed the tenets of the Declaration of Helsinki. Selenium and placebo were free and the results were provided for them. In cases where lipid abnormality was severe and the patients required multi-dimensional treatments (diet, supplements, and medication), necessary treatments were delivered after completion of the study under supervision of dietician and nephrologist. The study protocol was confirmed by the Ethics Committee of Kermanshah University. Written informed consent was obtained from patients. All data were kept confidential.

### Statistical analysis

The data were analyzed using the SPSS software for Windows (version 20). For description, mean and standard deviation (±SD) were used. To compare the data between experimental and control group, independent *t* test, paired *t* test, univariate analysis of variance, Wilcoxon signed rank test, and Mann-Whitney-U test were applied. Significant level of *P* was set at 0.05.

## Results

Seventy-four patients completed the study (36 in experimental group and 38 in control group). Mean (±SD) age of experimental and control groups was 58.03 (±13.04) and 60.09 (±14.27) years, respectively (*P*=0.524). There were 18 males (47.3%) in experimental group and 17 males (47.2%) in control group (*P*=0.642). Table 1 presents the baseline variables studied in both groups. As shown, except for selenium level and BUN, there was no significant difference between the two groups at baseline.

**Table 1:** Comparison of baseline characteristics between experimental (selenium supplementation) and control (placebo) groups

	**Experimental**	**Control**	***P***
BMI, kg/m^2^	24.18 (±3.86)	23.35 (±4.58)	0.40
SBP, mm Hg	140.69 (±24.78)	140.79 (±20.18)	0.98
DBP, mm Hg	83.17 (±16.72)	86.18 (±11.99)	0.37
Total cholesterol, mg/dl	163.11 (±58.12)	148.29 (±32.87)	0.17
LDL-C, mg/dl	85.66 (±31.12)	80.55 (±21.13)	0.41
HDL-C, mg/dl	37.26 (±17.41)	33.95 (±8.37)	0.78
Triglyceride, mg/dl	153.22 (±78.77)	145.32 (±73.2)	0.70
Selenium	34.33 (±12.66)	44.05 (±26.17)	0.04
BUN, mg/dl	123.97 (±38.11)	104.58 (±37.56)	0.03
Creatinine, mg/dl	7.55 (±2.51)	7.52 (±2.33)	0.95

Abbreviations: BMI, body mass index; SBP, systolic blood pressure; DBP, diastolic blood pressure; LDL, low-density lipoprotein; HDL, high-density lipoprotein; BUN, blood urea nitrogen All data are expressed as mean (±standard deviation).

Mean (±SD) serum LDL-C level significantly increased in experimental group from 85.66 (±31.12) to 109.12 (±32.29) mg/dl (*P*<0.001). Likewise, in control group LDL-C significantly increased from 80.55 (±21.13) to 97.05 (±28.07) (*P*<0.001). However, with control of LDL-C level before the study, it was revealed that LDL-C difference was not significant (*P*=0.21). Total cholesterol difference did not show significant change before and after the study in any group. With control of baseline value, it did not have significant change (*P*=0.95). Triglyceride level significantly decreased after the study in both groups (*P*<0.001). After controlling of the baseline value of triglyceride, it was revealed that there was no significant difference between two groups (*P*=0.37; Table 2).

**Table 2:** Comparison of studied variables between experimental (selenium supplementation) and control (placebo) groups after three months

	**Experimental**	**Control**	***P***
BMI, kg/m^2^	24.21 (±3.85)	23.35 (±4.58)	0.45
SBP, mm Hg	141.97 (±21.54)	144.08 (±19.79)	0.40
DBP, mm Hg	85.03 (±11.79)	85.50 (±10.88)	0.24
Total cholesterol, mg/dl	160.47 (±44.08)	151.79 (±27.18)	0.95
LDL-C, mg/dl	109.12 (±32.29)	97.05 (±28.07)	0.21
HDL-C, mg/dl	36.11 (±5.80)	36.34 (±8.65)	0.23
Triglyceride, mg/dl	113.94 (±82.08)	98.27 (±65.36)	0.73
Selenium	180.97 (±40.60)	53.63 (±36.72)	<0.001
BUN, mg/dl	114.08 (±31.43)	99.62 (±33.52)	0.24
Creatinine, mg/dl	10.14 (±14.01)	8.57 (±2.02)	0.23

Abbreviations: BMI, body mass index; SBP, systolic blood pressure; DBP, diastolic blood pressure; LDL, low-density lipoprotein; HDL, high-density lipoprotein; BUN, blood urea nitrogen All data are expressed as mean (±standard deviation).

## Discussion

We studied the effects of selenium supplementation in hemodialysis patients who suffered from low serum levels of selenium. We observed a significant difference of serum selenium among the patients who used selenium and the group that did not receive any selenium. Therefore it could be concluded that in hemodialysis patients whose levels of selenium is low, supplemental selenium raise their serum selenium level. This is comparable to a former study which reported that intravenous supplemental selenium (400 mg) two times per week for two months improved the oxygen radical scavenger system and increases selenium concentrations in plasma and erythrocytes and the activity of selenium dependent glutathione peroxidase (16).

Pakfetrat et al (15) studied 35 hemodialysis patients, 34 continuous ambulatory peritoneal dialysis (CAPD) patients in addition to a healthy group for a period of more than 3 months. The results suggest that selenium level in hemodialysis patients was significantly higher compared to CAPD patients, and in comparison to the healthy group the levels are significantly lower in these patient groups. Therefore, CAPD patients are at higher risk of effects of lower selenium levels than the hemodialysis patients. Also, Chen et al (12) in a study conducted on 81 hemodialysis patients and 42 healthy individuals found out that the levels of selenium in patients with chronic renal failure are significantly lower than in healthy individuals which are consistent with the findings of our research. However, given the differences in the selenium levels between the two groups, there was no significant difference between their levels of LDL-C.

Also, in another study conducted by Martí del Moral et al (13) on 117 hemodialysis patients for a period of two years, they found that the levels of selenium in dialysis patients were significantly lower than selenium levels in the individuals in the controlled group, and it could be an effective element in risk factors for atherosclerosis. Since the increase in lipids leads to atherosclerosis in hemodialysis patients, thus any association between selenium and lipid lowering offsetting a reduction of LDL-C and triglycerides have not been found in our study. Also, consumption of other antioxidants such as vitamins C and E and their effects on lipid oxidation levels could be another factor in cause changes in blood lipid levels in hemodialysis patients. Likewise, in a study conducted by Taccone-Gallucci et al on 103 hemodialysis patients and 39 healthy individuals concluded that serum concentration of selenium levels in hemodialysis patients who are treated with statins are higher than that in hemodialysis patients who were not treated with statins (11).

## Conclusion

Selenium supplementation in hemodialysis patients does not help reducing the harmful blood lipids levels as well as lowering LDL-C and cholesterol. And yet, contradictory results of other research studies show that it is imperative to conduct further similar studies on the effects of control of diet of patients in bigger population.

## Limitations of the study

In this project, there were some limitations including 1) limitation in the number of patients interested in participating in the study and taking selenium, 2) migration of the patients, and 3) the probability of mortality at older ages among patients undergoing chronic hemodialysis or lack of regular drug use in older age. Therefore, to avoid such limitations, larger sample should be considered before starting the study.

## Suggestions

The research team, in order to achieve the best results regarding the effects of selenium functions on the items discussed in the text, offers the following suggestions: 1) To perform studies with larger sample size, and 2) Long-term follow-up of patients in order to review and confirm or reject the effects of selenium on the studied items.

## Acknowledgments

We thank Mr. Masoud Ahmadi-Masoul and Ms. Neda Saedpanah, the staff nurse of the hemodialysis center and also Imam Reza hospital for their kind contribution to this study.

## Authors’ contribution

All authors contributed to design of the research and all authors have read, revised, and approved the final manuscript.

## Conflicts of interest

The authors declared no competing interests.

## Ethical considerations

Ethical issues (including plagiarism, misconduct, data fabrication, falsification, double publication or submission, redundancy) have been completely observed by the authors.

## Funding/Support

This manuscript extracted from MSc thesis 92236 that supported financially by deputy of research of Kermanshah University of Medical Science. The authors are grateful to all who helped in conducting the present Research.
